# Effective Combinations of Immunotherapy and Radiotherapy for Cancer Treatment

**DOI:** 10.3389/fonc.2022.809304

**Published:** 2022-02-07

**Authors:** Siting Yu, Yang Wang, Ping He, Bianfei Shao, Fang Liu, Zhongzheng Xiang, Tian Yang, Yuanyuan Zeng, Tao He, Jiachun Ma, Xiran Wang, Lei Liu

**Affiliations:** ^1^ Department of Head and Neck Oncology, West China Hospital, Sichuan University, Chengdu, China; ^2^ Laboratory of Aging Research and Cancer Drug Target, State Key Laboratory of Biotherapy, National Clinical Research Center for Geriatrics, West China Hospital, Sichuan University, Chengdu, China

**Keywords:** cancer immunity, immune tolerance, immunotherapy, radiotherapy, nanomaterials, drug-delivery system, radiosensitizers

## Abstract

Though single tumor immunotherapy and radiotherapy have significantly improved the survival rate of tumor patients, there are certain limitations in overcoming tumor metastasis, recurrence, and reducing side effects. Therefore, it is urgent to explore new tumor treatment methods. The new combination of radiotherapy and immunotherapy shows promise in improving therapeutic efficacy and reducing recurrence by enhancing the ability of the immune system to recognize and eradicate tumor cells, to overcome tumor immune tolerance mechanisms. Nanomaterials, as new drug-delivery-system materials of the 21st century, can maintain the activity of drugs, improve drug targeting, and reduce side effects in tumor immunotherapy. Additionally, nanomaterials, as radiosensitizers, have shown great potential in tumor radiotherapy due to their unique properties, such as light, heat, electromagnetic effects. Here, we review the mechanisms of tumor immunotherapy and radiotherapy and the synergy of radiotherapy with multiple types of immunotherapies, including immune checkpoint inhibitors (ICIs), tumor vaccines, adoptive cell therapy, and cytokine therapy. Finally, we propose the potential for nanomaterials in tumor radiotherapy and immunotherapy.

## Introduction

Radiotherapy (RT) is a standard treatment for malignant tumors. About 70% of tumor patients accept RT. RT causes irreversible damage to the DNA of tumor cells in the irradiation field to achieve local control of tumors. In the past decades, RT has made remarkable progress (1, 2). Recently, with a better understanding of tumor immune mechanisms, the application of immunotherapy such as immune checkpoint inhibitors (ICIs) (3), tumor vaccines (4), adoptive cell therapies (5), cytokine therapies (6), and other immunotherapies have increased. Immunotherapy has gradually been affirmed as the most likely direction to cure cancer in the field of tumor therapy by the medical community (7). However, radiation induces the release of myeloid-derived suppressor cells (MDSCs), M2-like tumor-associated macrophages (M2-like TAMs), T-regulatory cells (Tregs), N2 neutrophils, and immunosuppressive cytokines (TGF-β, IL-10) to promote the immunosuppressive microenvironment (8, 9). In addition, the presence of hypoxic cells in tumors leads to resistance to RT, which remains a hot topic in RT research. At the same time, due to the complexity of the mechanisms of tumor immune escape, in clinical practice, single immunotherapy is limited to a few tumors and can only benefit a few patients. With further research, especially the discovery of the abscopal effect induced by RT, it has been suggested that RT is closely influenced by the immune microenvironment. Many preclinical studies have shown that irradiation triggers immunogenic cell death (ICD), which promotes the release of tumor-associated antigens (TAAs), changes the tumor microenvironment (TME), and activates the immune system to exert an anti-tumor immune response (10, 11). Tumors with weak immunogenicity can benefit from RT combined with different types of immunotherapies. The synergy between immunotherapy and RT has become a hot field in oncology therapy.

**Figure 1:**
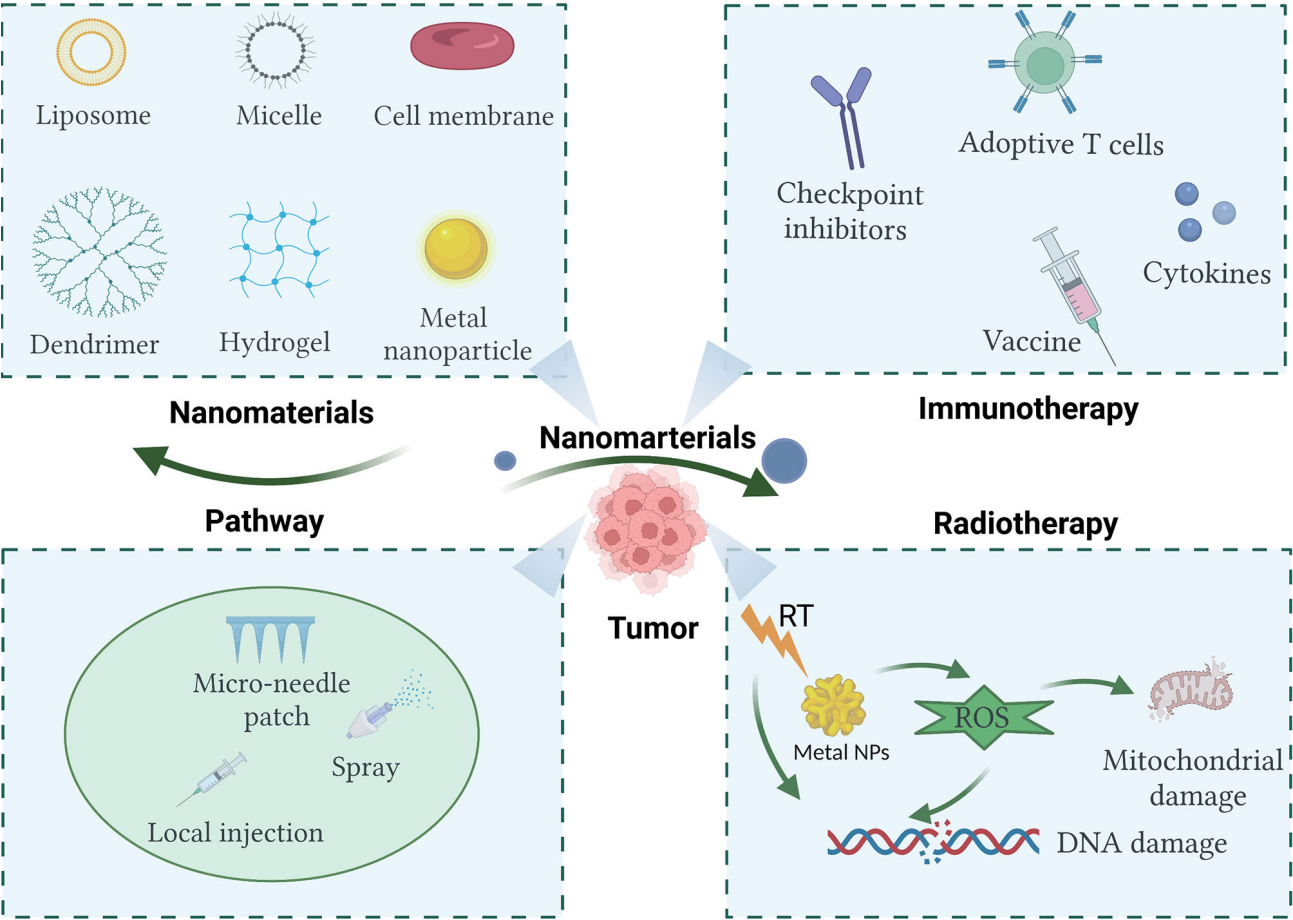
Nanomaterials used in cancer immunotherapy and radiotherapy show promise in enhancing therapeutic efficacy. Created with BioRender.com.

In recent years, with the rapid development of nanomaterials in biomedical applications, a significant number of studies have shown that multifunctional nanomaterials can deliver one or more immunomodulators and other drugs to tumor sites, which improves the immunosuppressive microenvironment (12, 13). Moreover, some multifunctional nanomaterials can be used as radiosensitizers to improve the efficacy of RT through photothermal effect, photokinetic effect, or direct regulation of hypoxia. Nanomaterials have great research value and clinical transformation prospects (14). Based on current literature, this review briefly discusses the mechanisms of cancer immunotherapy and RT, as well as the research progress of several immunotherapies combined with RT. It attempts to explore the challenges of synergistic treatment strategies for the two tumor treatment methods. Finally, the research progress of several kinds of nanomaterials in enhancing the effect of tumor immunotherapy and RT is reviewed (**Figure 1**).

## The Role of the Immune System in Anti-Tumor Activity

### Innate Immunity and Adaptive Immunity

The immune system exerts anti-tumor mechanisms through innate and adaptive immunity (15). The innate immune system, as the host’s first line of defense, exerts anti-tumor effects mainly by sensing pathogen-associated patterns (DAMPs) or danger-associated molecular patterns (DAMPs) (16). The recognition of PAMPs and DAMPs mainly rely on a series of pattern recognition receptors (PRRs), including Toll-like receptors (TLRs), RIG-I-like receptors (RLRs), Nod-like receptors (NLRs), AIM2-like receptor (ALRs), C-type lectin receptor (CLRs), and other DNA sensors (17, 18). PRRs recognize DAMPs and PAMPs, activate intracellular signaling cascades, and eventually lead to a series of immune responses. There are several myeloid cells involved in the innate immune responses, which include dendritic cells (DCs), monocytes, macrophages, polymorphonuclears, mast cells, and innate lymphoid cells (ILCs), such as natural killer cells (NKs) (19). Among them, DCs, macrophages, and NKs are the front-line cells of innate immunity. Normal cells can express major histocompatibility complex class I molecules (MHC I) on the cell surface, which inhibits NKs by acting on their Killer cell Ig-like Receptor (KIR). Tumor cells lack MHC-I molecules. NK cells lose their inhibitory signal when encountering MHC-I-deficient tumor cells. Thus, tumor cells are vulnerable to NK cell-mediated lysis (20). Macrophages exert cytotoxic effects through nonspecific phagocytosis.

Adaptive anti-tumor immune responses include humoral immunity and cellular immunity. In the humoral immune response, the immune system produces specific antibodies, thus exerting an anti-tumor effect. The mechanisms of tumor cell killing by antibodies can be summarized as the following: (1) The binding of the antibody and agonistic receptors to stimulate tumor immunity or effect tumor cell apoptosis (represented by the mitochondrion). The binding of the antibody and antagonist receptors can prevent dimerization, kinase activation, and downstream signaling, resulting in decreased proliferation and death (21). (2) Immune-mediated cell killing mechanisms (including, antibody-dependent cellular cytotoxicity (ADCC), complement-dependent cytotoxicity (CDC), and regulation of T cell function) (21, 22). (3) Antibody’s specific impact on tumor vasculature and stroma. Some antibodies can block the binding of tumor cell surface adhesion molecules and vascular endothelial cell surface adhesion molecules ligand, thereby preventing tumor cell growth, adhesion, and metastasis (23). The essential effector cells of cellular immunity are CD8+T cells (CTL) and CD4+ helper T cells (Th1). The former can specifically recognize the antigen peptide-MHC І complex presented by antigen presenting cells (APC), which can mediate direct cytotoxic effects through the release of perforin 1 (PRF) and granzyme B (24). The latter can recognize antigen peptide-MHC II complex presented by the APC and can release a great number of immunomodulatory cytokines to enhance the killing function of CTL, including interferon (IFN)-γ and interleukin-2 (IL-2) (25).

### Cancer-Immunity Cycle

**Figure 2:**
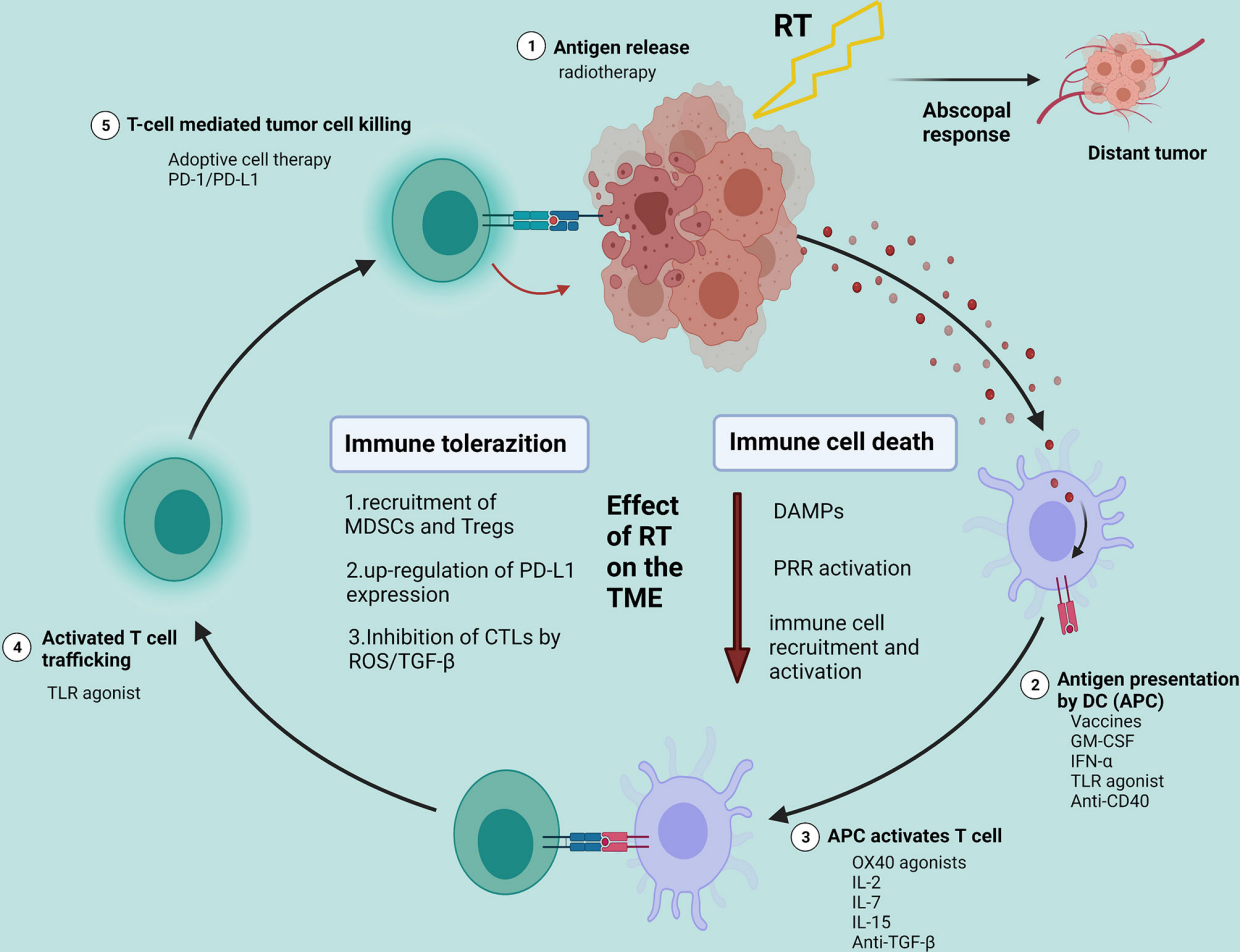
Effects of radiotherapy on the tumor microenvironment and potential strategies for the combination of radiotherapy with different immunotherapies. Created with BioRender.com. RT, radiotherapy; DAMPs, damage-associated molecular patterns; PRR, pattern recognition receptor; TME, tumor microenvironment; MDSCs, myeloid-derived suppressor cells; Tregs, T-regulatory cells.

A series of progressive events must be initiated to enable innate and adaptive immune responses to kill tumor cells effectively. This process is also defined as the Cancer-Immunity Cycle. This cycle can be divided into several main steps: the release of cancer cell antigens (step 1); cancer antigen presentation by dendritic cells (DC)/APCs (step 2); the priming and activation of effector T cell (step 3); trafficking of T cells to tumors (step 4); T-cell mediated tumor cell killing (step 5) (26, 27). The destroyed tumor cells release TAAs to further enhance immune responses. In the Cancer-Immunity Cycle, each step requires the coordination of several factors, including stimulatory factors and inhibitory factors. Each step can activate the entire immune cycle by strengthening the positive regulatory signal or inhibiting the negative regulatory to achieve treatment. In the first stage, RT and other therapies can cause ICD and initially activate the immune system. Tumor vaccines can promote cycle step 2. Anti-CTLA4 can primarily promote cycle step 3. VEGF inhibitors may enhance T cell infiltration into tumors—cycle step 4. CARs (chimeric antigen receptors) can promote step 5 (represented by CAR-T). Anti-PD-L1 or anti-PD-1 antibodies can primarily promote cycle step 5 (26) (**Figure 2**).

## Cancer Immunotherapy

At the end of the 19th century, William B. Coley, an American orthopedic surgeon, incidentally discovered an infection with *Streptococcus pyogenes* after surgery, and the patient’s cancer was relieved, which opened the door to immunotherapy for cancer for the first time (28, 29). Since then, many efforts have been attempted to better understand the critical role of the immune system in cancer progression. Immunotherapy, including ICIs, tumor vaccines, adoptive cell therapies, and cytokine therapies, has shown significant benefits in the treatment of various tumors, especially ICIs. The United States of America Food and Drug Administration (FDA) has approved PD-1/PD-L1 inhibitors to treat nine cancer types, including melanoma, non-small cell lung cancer, urothelial cancer, renal cell carcinoma, microsatellite instability or mismatch repair-deficient gastric cancer, colorectal cancer, hepatocellular carcinoma, and Merkel cell carcinoma (30–33).

### Insufficiency of Single Agent Immunotherapy

Some problems still exist in tumor immunotherapy. Firstly, only single immunotherapy is inefficient. Only 10% to 30% of patients respond to ICIs, and this is related to the complexity of the cancer cell immune system and the TME. Some studies have classified tumors based on the presence or absence of tumor-infiltrating lymphocytes (TILs) into the so-called cold and hot tumors. In short, hot tumors are tumors with infiltrating TILs, while cold tumors do not present any TILs. Tumors, such as melanoma and renal cell carcinoma, are more immunogenic and whose high levels of T cell infiltration are fertile ground for single ICIs or combination therapy (34). Although triple-negative breast cancer has relatively strong immunogenicity, it is still considered a cold tumor with lower TILs-infiltration and low expression of immune markers and cannot obtain an effective anti-tumor immune response through single-drug immunotherapy (35). Secondly, compared with chemotherapy, immunotherapy can lead to immune-related adverse events, which may occur in all organs, such as neurologic toxicity, renal toxicity, ocular toxicity, cardiovascular toxicity, hematologic toxicity (36). Moreover, the resistance mechanisms of tumor immunotherapy need further study.

### Resistance Mechanisms of Immunotherapy

When malignant cells occur in the body, the body can eliminate malignant cells through the above innate and adaptive immune responses. However, during the process of cancer immunoediting, the immunogenicity of the tumor is altered, and the tumor cells can escape immunosurveillance in a variety of ways. The study of tumor immune resistance mechanisms helps researchers to identify and design new tumor immunotherapy methods. The mechanisms of immune resistance have not been fully understood, although several mechanisms have been proposed (37–42) (**Table 1**).

**Table 1:** The potential mechanisms of immune resistance.

Type		Mechanisms	Examples
Intrinsic mechanisms	Adaptive resistance	Activation of signaling pathways	PI3K WNT/β-catenin IFN-γ
		Upregulation of constitutive PD-L1 expression	
		Epigenetic variations	KDM5B
		Loss of tumor antigen expression	Low mutation burden
	Acquired resistance
		Downregulation of tumor antigen presentation	The function defects of: Proteasome subunits transporters MHC itself β-2-microglobulin (B2M)
		Changes of T cells functional phenotype	
		The mutations of tumor cells	JAK1/JAK2 B2M
Extrinsic mechanisms		Inhibitory immune checkpoints	CTLA-4 PD-1 LAG-3 VISTA
		T cell exhaustion and phenotypic changes	
		immunosuppressive cells	TAMs Tregs
		The release of cytokine and metabolite	CSF-1 Adenosine tryptophan metabolites

*1. Intrinsic mechanisms of primary or adaptive resistance to immunotherapy.*

1. Enhancement of the PI3K signaling pathway caused by activation of the MAPK pathway and loss of PTEN expression;
2. Continuous activation of the WNT/β-catenin signaling pathway;
3. Absence of the IFN-γ signaling pathway;
4. Upregulation of constitutive PD-L1 expression;
5. Other underlying mechanisms: Epigenetic changes in tumor cell DNA may lead to the expression of immune-related genes, thereby affecting antigen processing, presentation, and immune escape. Tumors with a high mutation load can present higher levels of new antigens, which can induce anti-tumor immune responses. However, in some tumors, the DNA mutation frequency and immunogenicity are low, which leads to the lack of a T cell response.

*2. Intrinsic mechanisms of acquired resistance to immunotherapy.*

1. T cells change their functional phenotype and lose their killing activity;
2. Downregulation of tumor antigen presentation: The function defects of proteasome subunits, transporters, MHC itself, and β-2-microglobulin (B2M) during antigen processing will lead to the failure of antigen presentation;
3. The tumor develops escape mutations: Mutations in JAK1/JAK2 may result in tumor escaping the antiproliferative effect of INF-γ. B2M is necessary for HLA class І folding and transporting to the cell surface, and its mutation will lead to the lack of CD8+ T cell recognition.

*3. Extrinsic mechanisms of resistance to immunotherapy.*

1. Expression of inhibitory immune checkpoints on T cells, including CTLA-4, PD-1, LAG-3, VISTA;
2. T cell exhaustion and phenotypic changes;
3. The existence of immunosuppressive cells in the TME: Tregs can inhibit the T cell response by secreting inhibitory cytokines or by direct cell contact. MDSCs can promote angiogenesis, tumor invasion, and metastasis. M2 macrophages can secrete inhibitory IL-10 and TGF-β, thereby inhibiting immune response and promoting tumor growth and metastasis;
4. The release of cytokines and tumor metabolites in the TME: Colony stimulating factor 1 (CSF-1) recruits tumor infiltrating myeloid cells (TIMs) that inhibit tumor immunity, including M2-like TAMs and MDSCs. Adenosine can inhibit T cell proliferation and cytotoxicity through A2A receptors on T cells. Tryptophan metabolites have a direct negative defect on effector T cell function.

## Cancer Radiotherapy

With improvements in the precision of RT, it has become a key treatment option in modern cancer management (43, 44). Radiation can destroy cells in the irradiated area. There are currently several advanced technologies, including intensity-modulated radiation therapy (IMRT), image-guided RT (IGRT), high dose rate (HDR), brachytherapy (BT), stereotactic ablative body radiotherapy (SABR), proton therapy, and carbon ion radiotherapy (CIRT) (45–47). Proton heavy ion therapy is currently internationally recognized as the most advanced and ideal tumor RT technology. It has high accuracy, strong killing power, and a wide application range in the treatment of tumors. Furthermore, proton heavy ion RT is a noninvasive treatment (48). With the advancement of heavy ion therapy equipment and technology, the decline of treatment costs, and the advancement of research, heavy ion therapy will gradually be popularized in various countries across the world.

### Immunological Effects of Radiotherapy

Traditionally, it is believed that the mechanism of RT is to cause irreparable DNA damage and induce different types of tumor cell death, such as apoptosis, necrosis, autophagy, mitotic catastrophe, and senescence. The most typical mechanism involves double-strand breaks (DSB) (49–51). However, increasing preclinical and clinical data shows that RT can affect not only the local tumor but also activate the immune system and induce ICD, which can explain the abscopal effect (45, 52).

**(1) Immunogenic Cell Death**

Chemical drugs and radiation can induce tumor cell apoptosis and up-regulate characteristic protein molecules on the surface of apoptotic cells. These characteristic protein molecules induce the maturity of DCs and activate tumor-specific CTLs to kill tumor cells. Apoptosis of tumor cells caused by this process is called immunogenic cell death (ICD) (53, 54). The immune signaling molecules involved in this process are called danger-associated molecular patterns (DAMPs), such as calreticulin (CRT), adenosine triphosphate (ATP), heat shock protein (HSP), and high mobility group protein B1(HMGB1) signaling molecules (55).

DAMPs mainly initiate specific anti-tumor immune responses through the following mechanisms: (1) DAMPs are released and lead to cell death and the production of cellular antigens; (2) DAMPs stimulate the maturity of immature DCs and improve the ability of DCs to recognize tumors and present antigens;(3) Mature DCs activate tumor attack by specific CTLs. During this process, DCs provide three signals to activate T cells: (1) *via* the formation of the antigenic peptide/MHC-I and antigenic peptide/MHC-II complex for recognition by CD8+T cells and CD4+T cells, respectively; (2) by the activation of naive T cells and costimulatory receptors on the surface of DCs, such as CD80, which is necessary for effective T cell activation; (3) *via* additional polarization and differentiation signals delivered from DCs, including IL-12 or type I IFNs that are crucial for T cell differentiation into T cell effectors (55–57). In summary, RT can lead to cancer cell necrosis through the above mechanisms, releasing plenty of antigens and attracting immune cells to chemotaxis to the tumor site to kill cancer cells.

**(2) Abscopal Response**

In 1953, Mole et al. (58) found that when the local tumor lesions were irradiated, the tumor lesions outside the irradiation target area were also reduced, and they proposed the concept of “Abscopal response”. To date, the abscopal response has been reported in a variety of solid tumors, including melanoma, renal cell carcinoma, breast cancer, and hepatocellular carcinoma. With further research on the anti-tumor immune mechanism of the body, it is now well known that there are two main reasons for the abscopal effect of RT: (1) the lethal dose of irradiation induces immunogenic cell death (ICD) and (2) non-lethal dose irradiation induces immunoregulatory effects on tumor cells. By changing the immunological phenotype of tumor cells and enhancing the function of T cells, the killing ability of tumor cells is increased. Studies have shown that radiation can improve the ability of CTL cells to recognize and kill tumors by increasing the expression of TAAs and MHC-I on tumor cells (59). In a study of innate and adaptive immune cells in the TME, Gajewski et al. emphasized that RT induces the activation of innate immune pathways (including TLRs, NLRs, and STING pathways), which can activate DCs and promote the proliferation of T cells (60).

### Insufficiency of Single Radiotherapy

Radiation alone cannot kill all cancer cells. When the radiation enters the body, two ionization effects are produced. One is a direct effect on molecular DNA. The second is an indirect effect. Radiation can ionize water molecules to produce H+ and OH-, which can directly act on DNA molecular chains in cancer cells to cause molecular chain rupture. This indirect effect requires oxygen. Therefore, some types of tumors in an anoxic state are not sensitive to radiation. Hypoxia in hypoxic tumors causes less DNA damage at the same dose of radiation compared with well oxygenated ones. Meantime, Hypoxia will lead to the activation of the HIF signaling pathway. The activation of HIF1 can affect the expression of hundreds of genes, including vascular endothelial growth factor (VEGF) and angiopoietin-1 (ANGPT1), which can promote tumor survival (61). It also drives the expression of key enzymes in glycolysis, leading to the accumulation of lactic acid, pyruvate, antioxidants glutathione, and NADPH to limit DNA damage (62).

Moreover, changes induced by RT in the TME are very contradictory (63). RT enhances the recruitment of anti-tumor T lymphocytes to the TME by regulating adhesion molecules. Conversely, RT leads to the recruitment of MDSCs and Treg cells in TME and promotes immune tolerance to tumor cells (64). Studies have also shown that RT can increase the expression of PD-L1 in TME cells, especially DC cells, and the up-regulation of PD-L1 expression inhibits CTLs activity both *in vitro* and *in vivo*, and thus promote tumor growth (65). Reactive oxygen species (ROS) produced by RT enhance the activation of TGF-β, which is a crucial barrier to inhibit RT-induced T cell response to various endogenous tumor antigens (66). Furthermore, precise RT techniques, such as IMRT, maximize tumor dose and minimize organ dose, reducing the toxicity of RT, but some patients still experience adverse reactions. The side effects of RT include short-term toxicity and long-term consequence. Short-term adverse reactions occur during treatment or within 3 months after RT, such as mucositis, which is usually cured in weeks to months. Late effects, such as fibrosis, are usually considered irreversible. The early and late toxicity of RT largely depends on targeted tissue, including acute gastrointestinal (GI) damage, cardiac toxicity, cognitive impairment, reproductive disorders, deformity and impairments to bone growth, hair loss (67). In addition to optimizing physical techniques, an obvious way to prevent adverse effects of RT is to reduce the radiation dose that affects normal tissues, such as the combination with other treatments.

## Combination Therapy Between Immunotherapy and Radiotherapy

As mentioned above, hypoxic cells in tumor tissue are resistant to RT, which is one of the reasons for tumor recurrence after radiotherapy. The low response rate of immunotherapy also needs to be improved. More and more clinical and preclinical studies have shown that RT and immunotherapy can be complementary in recent years. Radiotherapy can induce ICD, release tumor neoantigens (TANs), and stimulate anti-tumor immune effect *in vivo*. Cancer patients receiving RT showed distinct immunogenic patterns, which may improve the response of immunotherapies, especially when it is used in combination with immune-stimulating drugs such as immune checkpoint inhibitors (68). At the same time, immunotherapy may make the tumor more sensitive to RT, thus providing a unique benefit for local treatment of tumors.

To date, exploring the synergistic effects of RT and immunotherapy is a reasonable solution, and many preclinical and clinical studies have yielded some results in combination therapy for tumor control (69). At present, RT combined with immunotherapy mainly starts from several directions. One is to promote tumor antigen recognition and presentation, such as using GM-CSF, toll-like receptor agonists; The other is to eliminate the immunosuppressive factors in the tumor microenvironment, mainly by using immune checkpoint inhibitors, such as CTLA-4 inhibitors and PD-1/PD-L1 inhibitors. Other approaches are also emerging, such as adoptive cell therapy.

### Combination of Immune Checkpoint Inhibitor Therapy and Radiotherapy

ICIs treatment targeting programmed cell death receptor 1 (PD-1) and programmed cell death ligand 1 (PD-L1) have shown solid clinical efficacy (70, 71). The expression of PD-L1 is representative predictive biomarkers of PD-1/PD-L1 inhibitor response. At present, blocking antibodies against PD-1 or PD-L1 have been developed and approved for the treatment of various advanced cancers, including non-small cell lung cancer (NSCLC), which is the most successful ICIs application (72). Although this treatment can achieve long-lasting effects in some patients, the current trial data shows that more than half of the patients still fail to show a significant response to checkpoint blockers (71). For example, a large proportion of patients with advanced NSCLC are resistant to inhibitors, and only a small number of patients benefit from PD-1/PD-1 inhibitors (73). Therefore, other therapies need to be combined to enhance and drive anti-tumor responses in patients who currently do not respond. As mentioned above, the effects of RT on the immune system include activation and inhibition. The combination of SBRT and PD-1/PD-L1 inhibitors can enhance positive immune regulation. The enhanced expression of PD-L1 induced by SBRT can make patients more sensitive to subsequent PD-1/PD-L1 inhibitors (74).

The combination of RT and anti-PD-1/PD-L1 antibodies is a promising strategy supported by many preclinical and clinical evidence (9). Preclinical studies have shown that low-grade RT combined with PD-1/PD-L1 inhibitors can improve the survival rate of mouse models of melanoma, renal cell carcinoma, breast cancer, and NSCLC and prevent tumor recurrence (75–77). In 2013, Zeng et al. reported the combination of anti-PD-1 antibody and stereotactic radiation therapy (SRT) could improve the survival rate of mice with intracranial glioma (78). A secondary analysis of phase 1 KEYNOTE-001 trial suggested that patients with advanced NSCLC treated with RT had longer progression-free survival and overall survival than those who did not receive RT before pembrolizumab treatment (79). In a retrospective study, the data of 208 patients receiving stereotactic radiosurgery (SRS) or whole-brain radiation therapy combined with CPIs or BRAF/MEK inhibitors were analyzed. The results showed that patients treated with anti-PD-1+ anti-CTLA-4 or anti-PD-1 alone combined with SRS had the best survival rate, and the 12-month survival rates were 100% and 70%, respectively (80).

### Combination Therapy Between Tumor Vaccine and RT

The tumor vaccine has been a research hotspot in recent years. Unlike the mechanism of traditional vaccines for disease prevention, tumor vaccines aim to expand the response of tumor-specific T cells by enhancing active immunity, attacking the formed tumors, and achieving the purpose of removing or controlling tumors. It has long been considered as possible and practical cancer immunotherapy (4, 81). According to the delivery method of antigen, tumor vaccines are mainly divided into peptide/protein vaccines, cell vaccines (such as tumor cell vaccines, DC vaccines, and engineered cell vaccines), nucleic acid vaccines (such as DNA vaccines and RNA vaccines) and viral vector vaccines (82, 83). At present, although hundreds of therapeutic tumor vaccines are in the clinical evaluation stage, the US FDA has only approved three therapeutic tumor vaccines:(1) Bacillus Calmette-Guerin (BCG), used for non-muscle-invasive bladder cancer (NMIBC); (2) DC vaccine (Sipuleucel-T), used for metastatic castration-resistant prostate cancer; (3) Talimogene laherparepvec (T-VEC), used to treat advanced melanoma. The three vaccines were approved based on their superior survival rates (Sipuleucel-T and T-VEC), more prolonged disease-free survival (BCG), and sustained efficacy (T-VEC) (84). Among them, Sipuleucel-T is the FDA-approved tumor vaccine for therapeutic purposes, which is a landmark breakthrough in the field of tumor therapeutic vaccines (85).

Vaccines alone are not sufficient to induce an immune response strong enough to eradicate tumors. Effective anti-tumor response requires the synergy of CD4+ T cells, tissue-resident memory T cells (TRM), and other immune cells. However, most vaccines currently focus on inducing CD8+ T cells. There is evidence that the combination of cancer vaccine and RT can produce a synergistic effect, enhance the immune response *in vivo*. RT can up-regulate major histocompatibility complex (MHC), apoptosis-related receptors, soluble intercellular adhesion molecule-1 (ICAM-1), TAA and enhance vaccine-mediated tumor cell lysis. Low-dose RT can make tumor cells more sensitive to effector T cells (86–88). Currently, many trials that combine vaccines with RT are underway. According to the results of preclinical studies on mouse models, it was found that the combination of RT and DC-based vaccines pulsed with high hydrostatic pressure (HHP)-inactivate tumor cells could significantly retard tumor growth by generating a favorable tumor immune microenvironment (89). Wang et al. treated esophageal cancer patients with DC loaded with heat shock apoptotic esophageal cancer cell antigen combined with RT. The serum levels of IL-2, IL-12, and IFN-γ, and the proportion of CD8 + T cells were significantly higher in patients than in the baseline and RT alone groups at the 2-year follow-up. The 1-year and 2-year survival rates were also improved (90).

### Combination Therapy Between Adoptive Cell Therapy and RT

Adoptive cell therapy (ACT) is an individualized tumor treatment method. ACT can target antigen-specific tumor cells by isolating immunoreactive cells from patients, inducing differentiation, modification, and amplification *in vitro*, and then transfusing them back into patients. ACT mainly includes tumor infiltrating lymphocytes (TIL), Lymphokine-Activated Killer Cells (LAK), cytokine-induced killer (CIK), DC, NK, CAR-T, and TCR-T, among which CAR-T cell therapy is a hot research topic. CAR-T enables the patient’s T cells to express the chimeric antigen receptor (CAR) through gene transduction. The modified T cells are returned to the patient’s body to generate many CAR-T cells that specifically recognize tumors and kill tumor cells (91–93). CAR-T cells have a particular effect in treating solid tumors such as renal cell carcinoma, HER2 positive sarcoma, ovarian cancer (94), and colorectal cancer (95). In 2017, the US FDA approved for the first time two targeted CD19-targeted CART-cells: Tisagenlecleucel and Axicabtagene Ciloleucel for the treatment of childhood acute lymphoblastic leukemia and adult advanced sizeable B-cell lymphoma, respectively (96).

With the approval of four autologous CAR-T therapies targeting CD19 (Kymriah, Yescarta, Tecartus, and Breyanzi), the significant efficacy of CAR-T therapy has gradually been recognized in the industry (97). However, the response of CAR-T cells therapy in 90% of patients with solid tumors is still inadequate. Moreover, many patients relapse because tumors escape by antigen loss or modulation, and the immune memory is not guaranteed (98, 99). Therefore, promoting the migration of CART cells to tumor sites and improving the immunosuppressive microenvironment that may inhibit the function and persistence of CAR-T cells are very important for improving the therapeutic effect (100). Studies have shown that RT combined with CAR-T therapies may be more effective than single CAR-T therapies alone. The early results of preclinical data support the hypothesis that RT can promote CAR-T cell activity in solid tumors (101).

Preclinical studies have shown that the subtherapeutic dose of local RT combined with NKG2D-based CAR T cells can promote the migration of CAR-T cells to the tumor site, resulting in a synergistic effect in two independent syngeneic mouse glioma models (101). In the pancreatic cancer model with heterologous expression of sialyl Lewis-A (sLeA), the author found that not only sLeA^+^ but also sLeA^-^ tumor cells irradiated by low-dose radiation were also susceptible to CAR treatment, reducing the recurrence of antigen-negative tumors (102). Another study showed that BCMA-targeted CAR T-cell therapy plus RT for the treatment of refractory myeloma reveals offers potential synergies in patient outcomes (103).

### Combination Therapy Between Cytokine Therapy and RT

Cytokines are the main regulatory factors of innate and adaptive immunity. In the process of immunotherapy, cytokines directly stimulate immune effector cells in tumor sites, enhance cytotoxicity, and enable immune cells to communicate in a short distance (104). Through the study of animal tumor models, it was found that cytokines had extensive anti-tumor activities. IFN-α, GM-CSF, IL-2, IL-12, IL-15, and IL-21 were effective in various murine cancer models (105–108). At present, the US FDA has approved many cytokine drugs, such as IFN-α, which was approved for adjuvant treatment of stage III melanoma patients and several refractory malignant tumors. High-dose interleukin-2 (HDIL-2) was approved for the treatment of renal cell carcinoma and melanoma.

Due to its dose-limiting severe toxicity, cytokines have not achieved the efficacy prospect of preclinical trials in single-drug treatment. For example, although IL-2 therapy shows high cardiovascular, gastrointestinal, neurological, lung, liver, kidney, and hematological toxicity (109, 110), it has an essential auxiliary function and has great therapeutic potential in combination with other treatment strategies. Some studies on cytokine therapy combined with RT are ongoing. In the colon cancer model, radiation combined with L19-IL2 can cause systemic anti-tumor response and control tumor progression. This combination increases the phenotype of memory-like CD8+ T cells and prevents tumor recurrence (111). Demaria et al. reported that RT and adjuvant IL-15 produced the maximum anti-tumor response in breast cancer mouse models, which was attributed to increased tumor infiltration of NK, CD4+, and CD8+ T cytotoxic lymphocytes. In addition, TNF-α before RT has shown benefits. Cell colony-stimulating factor (GM-CSF) also showed benefits after RT.

## Application of Nanomaterials in Immunotherapy and Radiotherapy

At present, most immunotherapy drugs are biomacromolecule drugs. How to maintain the activity of these drugs, improve drug targeting, and reduce side effects is a problem that needs to be resolved. In addition, because of the abnormal tumor vascular structure and the excessive oxygen metabolism in tumor tissue, the TME is usually hypoxic. Hypoxic cells are not sensitive to radiation, leading to the failure of RT (112, 113). In recent years, the emergence of functional nanomaterials has brought new hope for tumor immunotherapy and RT. Nanomaterials are expected to overcome the limitations of traditional drug delivery systems, including low water solubility, low bioavailability, multidrug resistance, and non-specificity (114, 115). In addition, considerable research has shown that multifunctional nanomaterials can be used as RT sensitizers, which can make tumor local temperature rise and improve tumor hypoxia through photoelectric effect and photothermal effect, to improve the curative effect of RT (116–118).

### Targeted Drug Delivery System Based on Nanomaterials

Nano drug delivery systems (NDDs) are drug delivery systems of nanometer size (1–100 nm), a hotspot in the research of new drug delivery systems, with many advantages. First, they can be designed to protect therapeutic drugs until they are delivered to target cells, thereby reducing toxic side effects (119). Second, delivery systems can achieve spatiotemporal control of the treatment if they are responsive to stimuli such as pH, temperature, or light, thus keeping the cargo inactivated until it accumulates in the target cell (120, 121). Thirdly, due to the difference in carrier materials, some nano-delivery systems can increase the solubility of drugs, while others can control the release rate of drugs to achieve the effect of slow and controlled release (122). In 1995, researchers released the first liposome-based nanomedicine doxorubicin for cancer treatment. Currently, a variety of NDDs have been used in immunotherapy. Generally, drug carriers based on nanomaterials can be divided into three main groups: Organic carriers, inorganic carriers, and biomimetic nanocarriers (123, 124). Organic nanoparticles (NPs) as drug delivery systems mainly include: lipid-based NPs (liposomes), polymer-based NPs and micelles, synthetic low-density lipoprotein (LDL), dendrimers, biodegradable polyesters, albumin-based nanovectors, realgar NPs, polysaccharide polymer, citrus lemon-derived nanovesicles (123, 125). Inorganic NPs mainly include carbon NPs such as fullerenes, metallic NPs such as gold NPs, ceramic NPs, and other metal and nonmetal NPs (126). Biomimetic nanomaterials for drug delivery mainly use cell membranes such as red blood cell membrane, immune cell membrane, cancer cell membrane, and platelet membrane (127). To date, several clinical trials investigating immunotherapy-based on nanomaterials are underway (**Table 2**).

**Table 2:** Clinical trials about immunotherapy based on nanomaterials.

NCT number	Title	Cancer type	Treatment	Phase	Recruitment Status
NCT03719326	A Study to Evaluate Safety/Tolerability of Immunotherapy Combinations in Participants With Triple-Negative Breast Cancer or Gynecologic Malignancies	Breast Cancer	Drug: Etrumadenant Drug: IPI-549 Drug: Pegylated liposomal doxorubicin (PLD) Drug: nanoparticle albumin-bound paclitaxel (NP)	Phase 1	Active, not recruiting
NCT04751786	Dose Escalation Study of Immunomodulatory Nanoparticles (PRECIOUS-01)	Advanced Solid Tumor	Drug: PRECIOUS-01	Phase 1	Recruiting
NCT04249167	Cryoablation, Atezolizumab/Nab-paclitaxel for Locally Advanced or Metastatic Triple Negative Breast Cancer	Breast Cancer	Drug: Atezolizumab Procedure: Cryosurgery Drug: Nab-paclitaxel	Early Phase 1	Active, not recruiting
NCT02740985	A Phase 1 Clinical Study of AZD4635 in Patients With Advanced Solid Malignancies	Advanced Solid Malignancies Non-Small Cell Lung Cancer (NSCLC) Metastatic Castrate-Resistant Prostate Carcinoma (mCRPC) Colorectal Carcinoma (CRC)	Drug: AZD4635 Drug: Durvalumab Drug: Abiraterone Acetate Drug: Enzalutamide Drug: Oleclumab Drug: Docetaxel	Phase 1	Active, not recruiting
NCT02662634	A Safety and Feasibility Study of AGS-003-LNG for the Treatment of Stage 3 Non Small Cell Lung Cancer	Non-small Cell Lung Cancer (NSCLC)	Biological: AGS-003-LNG Drug: Carboplatin Drug: Abraxane Drug: Alimta Drug: Cisplatin Drug: Taxol Radiation: Radiation Therapy	Phase 2	Withdrawn
NCT04929041	Testing the Addition of Radiation Therapy to the Usual Treatment (Immunotherapy With or Without Chemotherapy) for Stage IV Non-Small Cell Lung Cancer Patients Who Are PD-L1 Negative	Lung Non-Small Cell Carcinoma	Drug: Carboplatin Biological: Ipilimumab Drug: Nab-paclitaxel Biological: Nivolumab Drug: Paclitaxel Biological: Pembrolizumab Drug: Pemetrexed Other: Quality-of-Life Assessment Radiation: Stereotactic Body Radiation Therapy	Phase 2 Phase 3	Not yet recruiting
NCT02530489	Nab-Paclitaxel and Atezolizumab Before Surgery in Treating Patients With Triple Negative Breast Cancer	Breast Adenocarcinoma	Drug: Atezolizumab Drug: Nab-paclitaxel	Phase 2 Phase 3	Active, not recruiting
NCT04940286	Gemcitabine, Nab-paclitaxel, Durvalumab, and Oleclumab Before Surgery for the Treatment of in Resectable/Borderline Resectable Primary Pancreatic Cancer	Pancreatic Adenocarcinoma	Biological: Durvalumab Drug: Gemcitabine Drug: Nab-paclitaxel Biological: Oleclumab	Phase 2	Not yet recruiting
NCT03361319	Combination Nab-paclitaxel (N-P) and Nintedanib or N-P and Placebo in Relapsed NSCLC Adenocarcinoma (N3)	Lung cancer	Drug: Vargatef Drug: Abraxane Other: placebo	Phase 1 Phase 2	Withdrawn
NCT03739801	MM-398 and Ramucirumab in Treating Patients With Gastric Cancer or Gastroesophageal Junction Adenocarcinoma	Gastric Adenocarcinoma	Drug: Liposomal Irinotecan Other: Quality-of-Life Assessment Other: Questionnaire Administration Biological: Ramucirumab	Phase 1 Phase 2	Withdrawn
NCT03907475	Durvalumab in Combination With Chemotherapy in Treating Patients With Advanced Solid Tumors, (DURVA+ Study)	Malignant Solid Neoplasm	Drug: Capecitabine Drug: Carboplatin Biological: Durvalumab Drug: Gemcitabine Hydrochloride Drug: Nab-paclitaxel Drug: Paclitaxel Drug: Pegylated Liposomal Doxorubicin Hydrochloride	Phase 2	Recruiting
NCT03181100	Atezolizumab With Chemotherapy in Treating Patients With Anaplastic or Poorly Differentiated Thyroid Cancer	Thyroid Gland Carcinoma	Drug: Atezolizumab Biological: Bevacizumab Drug: Cobimetinib Drug: Nab-paclitaxel Drug: Paclitaxel Drug: Vemurafenib	Phase 2	Recruiting
NCT04892953	Local Consolidative Therapy and Durvalumab for Oligoprogressive and Polyprogressive Stage III NSCLC After Chemoradiation and Anti-PD-L1 Therapy	Lung cancer	Drug: Carboplatin Biological: Durvalumab Drug: Gemcitabine Procedure: Local Consolidation Therapy Drug: Nab-paclitaxel Drug: Paclitaxel Drug: Pemetrexed Other: Quality-of-Life Assessment Other: Questionnaire Administration	Phase 2	Not yet recruiting
NCT03606967	Testing the Addition of an Individualized Vaccine to Nab-Paclitaxel, Durvalumab and Tremelimumab and Chemotherapy in Patients With Metastatic Triple Negative Breast Cancer	Breast Carcinoma	Drug: Carboplatin Biological: Durvalumab Drug: Gemcitabine Hydrochloride Drug: Nab-paclitaxel Biological: Personalized Synthetic Long Peptide Vaccine Drug: Poly ICLC Biological: Tremelimumab	Phase 2	Recruiting

In Organic carriers, liposomes have attracted much attention. The liposome is a suitable drug carrier with high encapsulation efficiency, good targeting, and low toxicity (128). Its structure is similar to the cell membrane so that hydrophilic therapeutic agents can be encapsulated in liposomes, and hydrophobic therapeutic agents can be encapsulated in lipid bilayers. The liposome can deliver drugs to the cytoplasm of the cell by fusing the liposome into the lipid bilayer of the cell (129). Adjuvant in the liposome is a promising adjuvant strategy for immunogenicity vaccines, and plenty of preclinical studies are being carried out. Li et al. designed a nanoliposome composite (IR-7-Lipo/HA-CpG) coated with multivalent immune adjuvant (HA-CpG) and a photothermal sensitive agent inside for tumor photothermal ablation and combined immunotherapy. Under 808 nm laser irradiation, IR-7-Lipo can induce tumor cell necrosis and release tumor-associated antigens (TAAs), while immune adjuvant can promote antigen presentation. The results showed that IR-7-Lipo/HA-CpG can significantly regulate tumor microenvironment. *Vivo* experiments also showed effective tumor eradication and metastasis inhibition in mice (130).

Inorganic nanomaterials have unique optical, electrical, and magnetic properties. Therefore, they usually have the effect of photothermal therapy (PTT) and photodynamic therapy (PDT) in tumor immunotherapy (131), which is a suitable drug carrier as well as tumor therapeutic agent. Zhang et al. developed a new type of gold nanoparticle (AuNP) for tumor photothermal and immunotherapy. The experimental results show that the high photothermal conversion efficiency and photostability of AuNP can significantly improve the therapeutic effect of tumor photothermal therapy. At the same time, the surface modification of AuNP endows the material with immune characteristics and finally achieves the combined treatment of tumor photothermal and immune activation to improve the therapeutic effect (132). Inorganic nanoparticles have become a research hotspot in the biomedical field in recent years. Some inorganic nanoparticles have been used in clinical practice, but the study of their toxicity *in vivo*, biological distribution, and removal methods is still a challenge.

In recent years, nanocarriers based on bionics have been designed, such as red blood cells, exosomes, pathogens, tumor cell membranes (133, 134). Compared to conventional nanomaterials, bionic nanomaterial have natural structures, therefore have vital targeting and good biocompatibility, can deliver drugs to target cells or tissues (127). Deng et al. constructed a type of nanoparticle (NK-NPS) disguised by the NK cell membrane, loaded with photosensitizer 4,4’,4’’,4’’’-(Porphine-5,10,15,20-tetrayl) tetrakis (benzoic acid) TCPP. Photodynamic therapy (PDT) combined with immunotherapy can not only eliminate primary tumors but also effectively inhibit the growth of distal tumors. Therefore, the engineered NK cell-like nanoparticles in this study can provide a general strategy for effective cell membrane immunotherapy (135). Chen et al. selected ZIF-8 nanoparticles to load therapeutic proteins to form biomimetic nanocarriers (MP) and then used natural extracellular vesicles (EVM) to encapsulate MP nanocarriers to assemble EMP nanotransporters. The strategy of wrapping extracellular vesicles can not only effectively protect proteins from the degradation of proteases in the blood and the phagocytosis of phagocytes, but also help to penetrate the barrier of the cell membrane (136).

### Radiosensitizers Based on Nanomaterials

Nanomaterials have been widely used to improve the efficacy of RT because of their good biocompatibility, inherent radiosensitivity, and physicochemical properties such as enhanced permeability and retention effect in tumor tissues (137). The mechanisms of these radiosensitizers to improve the efficacy of RT are as follows (138, 139): (1) Enhanced absorption of ionizing radiation energy by tumor cells; (2) Some photocatalytic semiconductor nanoparticles can promote the generation of ROS under X-ray excitation, thereby enhancing the effect of RT (2); (3) Deplete GSH in tumor and then overcome the GSH-associated radioresistance and thus improve the therapeutic of RT; (4) Adjust cell cycle/signal pathway to make tumor cells more sensitive to radiation.

At present, plenty of preclinical radiosensitizers based on nanomaterials are underway. Song et al. designed a liposome-loaded anti-CLTA4 antibody that encapsulated H_2_O_2_ enzymes and H_2_O_2_ for IRT. Under X-ray irradiation, H_2_O_2_ enzyme and H_2_O_2_ produce O2 to alleviate tumor hypoxia and enhance RT efficacy and release anti-CLTA4 antibodies to increase the infiltration of cytotoxic T lymphocytes into tumor tissues (140). Liu et al. prepared PLGA nanoparticles loaded with immune adjuvant R837 and catalase, and catalase decomposed hydrogen peroxide in the tumor, which could improve tumor hypoxia for tumor RT sensitization. Radiotherapy could trigger the immunogenicity death of cancer cells, and tumor fragments were used as TAAs. Under the action of nanoparticles loaded with the immune adjuvant, an anti-tumor immune response was stimulated and then combined with ICIs to inhibit the growth of distal tumors effectively (141). Gao et al. nitrosylated maytansinoid DM1, and then the obtained prodrug DM1-NO was loaded onto poly(lactide-co-glycolic)-block-poly (ethylene glycol) (PLGA-b-PEG) nanoparticles. Under irradiation, oxidative stress increased, leading to the cleavage of S-N bond and the release of DM1 and NO, both of which are potent radiosensitizers. DM1 leads to cell enrichment at the more radiosensitive G2/M phase. NO forms toxic free radicals such as peroxynitrite under irradiation. The synergistic effect of these two components enhances the radiotherapy outcomes (142). Currently, many clinical trials about radiosensitizers based on nanomaterials are also in progress (**Table 3**).

**Table 3:** Clinical trials about radiosensitizers based on nanomaterials.

NCT number	Title	Cancer type	Treatment	Phase	Recruitment Status
NCT04899908	Stereotactic Brain-directed Radiation With or Without Aguix Gadolinium-Based Nanoparticles in Brain Metastases	Brain Cancer Brain Metastases Melanoma Lung Cancer Breast Cancer Colorectal Cancer Gastrointestinal Cancer	Radiation: Stereotactic Radiation Drug: AGuIX gadolinium-based nanoparticles Other: Placebo	Phase 2	Not yet recruiting
NCT03818386	Radiotherapy of Multiple Brain Metastases Using AGuIX^®^ (NANORAD2)	Brain Metastases	Drug: AGuIX^®^ Radiation: Whole Brain Radiation Therapy	Phase 2	Recruiting
NCT04094077	Evaluating AGuIX^®^ Nanoparticles in Combination With Stereotactic Radiation for Brain Metastases (NANOSTEREO)	Brain Metastases	Drug: AGuIX	Phase 2	Terminated
NCT04784221	Reirradiation by Nanoparticles and Hypofractionated Protontherapy of Relapsed Tumors: Non-randomized Phase II Study. (NANOPRO)	Recurrent Cancer	Radiation: Radiation by protontherapy associated to nanoparticles injection	Phase 2	Not yet recruiting
NCT04789486	Nano-SMART: Nanoparticles With MR Guided SBRT in NSCLC and Pancreatic Cancer	Lung Cancer Pancreatic Adenocarcinoma	Drug: AGuIX Radiation: Radiotherapy	Phase 1 Phase 2	Recruiting
NCT02820454	Radiosensitization of Multiple Brain Metastases Using AGuIX Gadolinium Based Nanoparticles	Brain Metastases	Drug: AGuIX Radiation: whole brain radiation therapy	Phase 1	Completed
NCT02721056	NBTXR3 Crystalline Nanoparticles and Stereotactic Body Radiation Therapy in the Treatment of Liver Cancers	Liver Cancer	Radiation: NBTXR3, IL or IA injection + SBRT	Phase 1 Phase 2	Terminated
NCT04240639	An Extension Study MRI/US Fusion Imaging and Biopsy in Combination With Nanoparticle Directed Focal Therapy for Ablation of Prostate Tissue	Neoplasms of the Prostate	Device: AuroShell particle infusion	Not Applicable	Recruiting
NCT02379845	NBTXR3 Crystalline Nanoparticles and Radiation Therapy in Treating Randomized Patients in Two Arms With Soft Tissue Sarcoma of the Extremity and Trunk Wall	Adult Soft Tissue Sarcoma	Device: NBTXR3 Device: Radiation therapy	Phase 2 Phase 3	Completed
NCT01433068	NBTXR3 Crystalline Nanoparticles and Radiation Therapy in Treating Patients With Soft Tissue Sarcoma of the Extremity	Adult Soft Tissue Sarcoma	Device: NBTXR3	Phase 1	Completed
NCT04484909	NBTXR3 Activated by Radiation Therapy for the Treatment of Locally Advanced or Borderline-Resectable Pancreatic Cancer	Pancreatic Adenocarcinoma	Other: Hafnium Oxide-containing Nanoparticles NBTXR3 Radiation: Radiation Therapy	Phase 1	Recruiting
NCT02680535	MRI/US Fusion Imaging and Biopsy in Combination With Nanoparticle Directed Focal Therapy for Ablation of Prostate Tissue	Neoplasms of the Prostate	Device: AuroShell particle infusion	Not Applicable	Not Applicable
NCT04505267	NBTXR3 and Radiation Therapy for the Treatment of Inoperable Recurrent Non-small Cell Lung Cancer	Lung Non-Small Cell Carcinoma	Other: Hafnium Oxide-containing Nanoparticles NBTXR3 Radiation: Radiation Therapy	Phase 1	Recruiting
NCT02033447	Magnetic Nanoparticle Thermoablation-Retention and Maintenance in the Prostate:A Phase 0 Study in Men (MAGNABLATE I)	Prostate Cancer	Other: Magnetic Nanoparticle Injection	Early Phase 1	Completed
NCT04862455	NBTXR3, Radiation Therapy, and Pembrolizumab for the Treatment of Recurrent or Metastatic Head and Neck Squamous Cell Cancer	Head and Neck Squamous Cell Carcinoma	Other: Hafnium Oxide-containing Nanoparticles NBTXR3 Radiation: Hypofractionated Radiation Therapy Biological: Pembrolizumab Radiation: Stereotactic Body Radiation Therapy	Phase 2	Recruiting
NCT04834349	Re-irradiation With NBTXR3 in Combination With Pembrolizumab for the Treatment of Inoperable Locoregional Recurrent Head and Neck Squamous Cell Cancer	Head and Neck Squamous Cell Carcinoma	Other: Hafnium Oxide-containing Nanoparticles NBTXR3 Procedure: Intensity-Modulated Proton Therapy Radiation: Intensity-Modulated Radiation Therapy Biological: Pembrolizumab	Phase 2	Recruiting

Therefore, the application of nanomaterials in the combined treatment of immunotherapy and radiotherapy can effectively improve the radiotherapy effect of lesion sites and can also load immunotherapy drugs to achieve collaborative treatment, which has become a new trend in tumor therapy. Ni et al. reported the design of two porous Hf-based nanoscale metal-organic frameworks (nMOFs). Hafnium oxide, as an effective radiosensitizer, not only has a radiosensitizing effect, but also causes ICD. The combination of hafnium oxide and anti-PD-L1 antibody can significantly elicit systemic anti-tumor immunity and produce good anti-cancer effects (143). Patel et al. successfully synthesized the complex, a multifunctional bacterial membrane-coated nanoparticle (BNP) composed of an immune-activating PC7A/CpG polyplex core coated with bacterial membrane and imide groups. BNP is used in combination with RT for *in situ* vaccination. Radiotherapy promotes TAAs release. Bacterial membrane captures tumor-related antigen and promotes DC uptake. Immunoadjuvant, PC7A/CpG polyple, further enhances antigen presentation of dendritic cells. The combination of BNP and RT can significantly eliminate tumors and induce tumor-specific anti-tumor immune memory (144).

However, there are still many challenges in conversion to future clinical application. In terms of biological safety, the physical effects of nano-systems should be evaluated, including biocompatibility, long-term toxicity, biodegradation. In terms of efficacy, nanocarriers’ size, morphology, chemical composition, and surface properties should be optimized to ensure the efficient accumulation of tumors. In addition, there are still some challenges in biomedical applications on how to achieve optimal radiation energy accumulation at significantly low radiation doses and minimize systemic side effects.

## Prospects and Challenges of Combined Immunotherapy With Radiotherapy

Although some clinical studies have confirmed the phenomenon of local RT combined with immunotherapy to stimulate abscopal response, the optimal combination strategy of RT combined with immunotherapy still needs to be further explored (145–147), including: (1) What are the best sequences to follow when combing RT with immunotherapy? Different studies have shown that the optimal timing of immunotherapy combined with RT is different, which is still controversial at present. IMMUNOBrainZH, a multicenter retrospective study in France, showed that patients with NSCLC intracranial metastases treated with nivolumab at intervals of less than three months were better than those treated at intervals of more than three months, with intracranial remission rates of 30.0% and 6.7% (148). This study suggested that early interventional immunotherapy after RT has a better clinical effect. However, a retrospective study reported at the 2019 American Society of Clinical Oncology (ASCO) meeting showed that patients with stage IV NSCLC received immunotherapy at least three weeks after SBRT, whose OS had obvious advantages compared with earlier immunotherapy (median OS was 19 months versus 15 months, P = 0.0335) (149). (2) What are the optional fraction and dose selection? Conventional fraction RT and large fraction RT are commonly used in clinical at present. Which fraction RT can maximize the abscopal response and further enhance the tumor-specific immune effect is still controversial. In the mouse model of prostate cancer, researchers found that high-dose fraction RT combined with PD-1 antibody immunotherapy was more effective in reversing the immunosuppressive microenvironment and controlling primary and distal metastatic tumors than conventionally fractionated radiotherapy (64). However, a study has also found that conventional fractionation RT combined with PD-L1 blockers can enhance the cytotoxicity of cytotoxic T cells and overcome the immune tolerance mediated by conventional fraction RT (150). (3) How to select the best immunotherapy to combine with RT? Single immunotherapy combined with RT may have the risk of drug tolerance. Therefore, the optimal combination of multiple immune-directed strategies and RT may be required to maximize the systemic anti-tumor immune activation (146). (4) The normal tissue toxicity and safety of the combination therapy. It has been reported that patients treated with both ICI and SBRT can develop radiation pneumonia, even though it is not clear whether SBRT or combined with ICI can enhance radiation pneumonia (151). Liu et al. reported three patients who received RT before anti-PD-1 treatment and developed pneumonia after four cycles of anti-PD-1 treatment (152). (5) What biomarkers are beneficial for deciding on the fine candidates for immuno-radiotherapy? Selecting appropriate biomarkers to determine which patients will benefit from combination therapy may help predict the clinical outcomes of patients receiving radioimmunotherapy (153). (6) How to increase the number of systemic anti-tumor reactions to an effective abscopal response? Dewan et al. carried out a preclinical study of large fraction RT combined with anti-CTLA-4 antibody in breast cancer. The study found that the group of 8 Gy × 3 times and 6 Gy × 5 times dose had abscopal response, and the former effect was more obvious, while the group of 20 Gy × 1 time did not observe abscopal response (154).

However, the number of preclinical and clinical studies on the combination of immunotherapy and RT continue to increase. In 2016, a study presented 93 activity trials that combined RT with inhibition of CTLA-4, PD-1, and PD-L1, transforming growth factor β -cytokines, or other immune molecules (155). In 2021, a random trial presented that adding RT to pembrolizumab immunotherapy significantly increased responses and outcomes in patients with metastatic non-small-cell lung cancer (156). A pooled analysis of two randomized trials showed that SBRT and pembrolizumab combined with trametinib were a new potential treatment option for patients with locally recurrent pancreatic cancer following surgical resection (157). If the results of more and more clinical trials are positive, it will determine how best to integrate these models and optimize synergy.

## Conclusion

To date, an increasing number of immunotherapy methods have been applied to the clinic, and a considerable amount of literature has reported the research results of RT combined with immunotherapy. However, large-scale clinical data are still limited about the combination of RT with various types of immunotherapies. How to reduce the side effects of combination therapy and how to find the optimal combination strategy of RT and immunotherapy (the optimal dose segmentation pattern sequential or synchronous) still needs to be further explored. At the same time, majority of drug delivery systems based on nanoparticles are still in the early stages of development, and their clinical application is still challenging. However, with the continuous development of nanotechnology, nanomaterials will be better used in tumor treatment and will achieve more favorable outcomes soon.

## Author Contributions

SY, YW, and PH contributed equally to this manuscript. BS, FL, ZX, TY, YZ were responsible for guiding the writing of the paper. TH, JM, and XW were responsible for the collection of literature materials. LL is responsible for the overall revision. All authors contributed to the article and approved the submitted version.

## Conflict of Interest

The authors declare that the research was conducted in the absence of any commercial or financial relationships that could be construed as a potential conflict of interest.

## Publisher’s Note

All claims expressed in this article are solely those of the authors and do not necessarily represent those of their affiliated organizations, or those of the publisher, the editors and the reviewers. Any product that may be evaluated in this article, or claim that may be made by its manufacturer, is not guaranteed or endorsed by the publisher.
